# Ant-mealybug mutualism modulates the performance of co-occurring herbivores

**DOI:** 10.1038/s41598-019-49334-3

**Published:** 2019-09-10

**Authors:** Chong Xu, Jia Su, Xiaobin Qu, Aiming Zhou

**Affiliations:** Hubei Insect Resources Utilization and Sustainable Pest Management Key Laboratory, College of Plant Science and Technology, Huazhong Agricultural University, Wuhan, 430070 China

**Keywords:** Behavioural ecology, Entomology

## Abstract

Mutualism between ants and honeydew producing hemipterans has been extensively studied. However, little is known on how ant-hemipteran mutualism impacts the co-occurring herbivores, which in turn affect the mutual relationship in ecosystems. Herein, we investigated the effect of ant-mealybug mutualism on the oviposition preference and spatial distribution of cotton leaf roller *Sylepta derogata*, a polyphagous herbivore, and in *Apantetes derogatae* performance, a larvae parasitoid of *S. derogata*. Leaf rollers constructed shelters for mealybugs to prevent them from enemy attack and preferred to lay eggs on plants with ant-mealybug mutualism. Egg abundance on mutualism-present plants was higher than on mutualism-absent plants. Leaf roller parasitoid *A. derogatae* showed higher parasitism on mutualism-absent plants. No obvious change in leaf roller egg abundance was observed when *A. derogatae* was excluded, suggesting that the parasitic pressure can also regulate the oviposition behavior of *S. derogate*. *Apantetes derogatae* showed higher aggressiveness in parasitizing leaf roller larvae at the absence of the mutualism. There was a definite correlation between leaf roller egg abundance and the number of patrolling ants on plants. Without ant-mealybug mutualism, *S. derogata* eggs showed a significantly aggregated distribution pattern, but a uniform distribution pattern was observed when the mutualism was present. Ant workers showed a consistently uniform distribution on plants. The results reveal a novel mediation effect of ant-mealybug association on the composition and structure of food webs in cotton field, which may contribute to a better understanding of the cascading effects of ant-hemipteran mutualism on other niche-related species in ecosystem.

## Introduction

Mutualistic interactions among species play important roles in mediating the dynamics and diversity of niche-related community^1,2^. The mutualism between ant and honeydew-producing hemipterans has been well documented in multiple ecosystems^3–5^, based on the defensive and aggressive activity of ants on sympatric herbivores by providing nutritious carbohydrate, which may severely interfere with the balance and stability of ecological communities^6,7^. Early work has exposed the positive and reciprocal effects of mutualism on the colonization and development of both species involved in the mutual interaction^8,9^. Currently, some studies have illuminated the ecological influence of ant-hemipteran mutualism on other co-occurring community members^6,10,11^. Ant diversity and aggressiveness can directly modulate the effects of the mutualism on the surrounding community^12^, affecting the ambient feed groups composed by predators and other arthropod specie^6^, in which of the abundance of arthropod species significantly decrease on trees with ant-hemipteran mutualism^13^.

The context-dependent effects of ant-hemipteran mutualism may directly or indirectly mediate involved community structure and population ecology^14,15^. Mutualism definitely facilitates the colony growth of ants and hemipterans^16–19^. Ant-hemipteran mutualism usually negatively affects the predators and parasitoids of hemipterans by excluding them from the host^20–22^. Fire ant-aphid interaction negatively affects the flower-visiting behavior of insects on rapeseed *Brassica napus*, e.g. the cabbage white butterfly *Pieris rapae* (L.) (Lepidoptera: Pieridae) spends much less time visiting the rapeseed plants with fire ant-aphid mutualism^23^. Fire ant-cotton aphid mutualism can indirectly improve cotton reproduction by consuming the damaging herbivores in cotton^24^. However, fire ant-aphid interaction also has unfavorable impacts on the yield of mung bean plant^25^. Interaction between Argentine ant and cotton aphid decreases the floral visitation frequency of honey bees and reduces plant reproduction by disrupting the pollination^26^. The reduction of pollination services caused by ant floral visitation in ant-hemipteran association may largely transform and disrupt the structure and stability of plant community. More importantly, ant-hemipteran mutualism may exacerbate this negative effect of floral visitation by ants on local community stability^26,27^. Although ant-hemipteran mutualism is ubiquitous and its effects has attracted increasing research attention, the complex and multiple influences of ant-hemipteran interaction on local community remain incompletely understood. Exploration of the interaction-mediated reciprocal effects on the composition and structure of food webs in associated community is critical to revealing the ecological outcomes of ant-hemipteran interaction in ecosystems.

*Phenacoccus solenopsis* (Tinsley), (Hemiptera: Pseudococcidae), a polyphagous invasive mealybug in China, has caused serious ecological consequences and economic losses for local agriculture^28^. Although several studies have elaborated the mutualism between ghost ant *Tapinoma melanocephalum* (Fabricius) and *P*. *solenopsis*^29–31^, few experimental studies have evaluated the broad ecological effects of this mutualism on local arthropod community. Cotton leaf roller, *Sylepta derogata* (Fabricius) (Lepidoptera: Pyralidae), is also a phytophagous lepidopteran with a wide host range including cotton *Gossypium hirsutum*, hibiscus *Hibiscus rosa-sinensis*, aubergine *Solanum melongena*, okra *Abelmoschus esculentus* and cowpea *Vigna unguiculata* in China^32^. *Apantetes derogatae* (Hymenoptera: Braconidae) is an important endoparasitoid of *S. derogate* larvae, especially for the 2^nd^ instar larvae^33,34^. Interestingly, *P*. *solenopsis* tends to utilize the curled leaves constructed by leaf roller larvae as shelters to protect itself from the *Aenasius bambawalei* (Hymenoptera: Encyrtidae), the most dominant and aggressive parasitoid on *P*. *solenopsis* in china^28,35^. When ant-mealybug mutualism is prevalent on *H*. *rosa-sinensis*, mealybugs greatly benefit from the protective shelters in the plants^35^. However, little is known about whether cotton leaf roller colony benefits from the ant-mealybug mutualism, and how this mutualism influences the prevalence of leaf roller. Herein, we conducted field and laboratory experiments to test the effects of ant-mealybug mutualism on the oviposition preference, parasitism and spatial distribution of *S*. *derogate*. Our results provide important insights into the trophic cascading effects of ant-hemipteran mutualism on the involved arthropod species. More generally, these experiments provide a new perspective for understanding the effects of ant-hemipteran interactions on local niche-related community and ecosystem.

## Plants and Insects

Cotton plants were grown from seeds (Jimian 11, non-transgenic, Academy of Agriculture and Forestry Sciences, Hebei, China) by cultivation soil (organic matter ≥20%; Jiangsu Peilei Technology Development CO., LTD) in plastic flowerpots (14 × 10 × 15 cm). *Phenacoccus solenopsis* nymphs and *S*. *derogata* adults were collected from cotton field in Huazhong Agricultural University and transferred to cotton plants (35 cm in height with 15–20 true leaves.). All insect colonies were reared for several generations in the laboratory at the temperature of 28 ± 2 °C and relative humidity of 60–70%. Leaf roller parasitoid *A*. *derogatae* and mealybug parasitoid *A*. *bambawalei* were also collected from the cotton field. Parasitoids were reared on leaf roller larvae and mealybug nymphs respectively in the laboratory at 28 ± 2 °C with a LD 16:8 h photocycle. *Tapinoma melanocephalum* colonies were collected from the suburb of Wuhan and were separated from the soil by dripping water into the plastic boxes until the colonies floated^29^. Ants were transferred and reared in plastic boxes and supplied with distilled water. The colonies were divided into several small sub-colonies (1.0 g) by weight using a microbalance (Sartorius BSA 224 S, Elk Grove, Illinois, USA). Each sub-colony included one queen and 1.0 g workers and was placed in a 9-cm plastic Petri dish as an artificial nest. Ant colonies were supplied weekly with live mealworm larvae (*Tenebrio molitor* L.; Coleoptera: Tenebrionidae) and 50 mL 10% honey water solution. All ant colonies were reared in the laboratory at the temperature of 28 ± 2 °C and relative humidity of 60–70%.

## Experiment Design

### Effect of ant-mealybug mutualism on oviposition preference of leaf roller

A completely randomized design was used to evaluate the effect of ant-mealybug mutualism on the oviposition preference of leaf roller in the cotton field of Huazhong Agricultural University (30°28′25″N, 114°21′22″W; elevation, 30 m). Four treatments were established, including (1) mutualism present (plant with ant-mealybug association) (n = 66), (2) mutualism absent (only ants patrolling on plant) (n = 66), (3) mutualism absent (only mealybug infection on plant) (n = 65), and (4) control (no ants and mealybugs) (n = 65). To minimize the differences in host plant quality, uniform and undamaged plants were randomly assigned to different experiments subsequently (plants with similar size and growth, 90–100 cm in height). The 3^rd^ instar nymph mealybugs were collected into a small centrifuge tube. The tubes, each containing 60 individuals, were placed on the top branches of each cotton plant. Then the nymphs crawled out from the tubes and began sucking the tender plant leaves. The colonization rate of mealybugs was checked after the inoculation. New mealybug colonies would be re-inoculated if the initial inoculation did not result in a satisfying colonization rate. The ghost ant *T. melanocephalum* was observed as a dominant native ant which tended mealybugs and other hemipterans. In ant-excluded plants, paraffin was applied to the base trunk of the plants to discourage ant approach. The percentage of leaf roller infection and number of eggs produced by *S*. *derogata* on each plant were recorded after one week.

Further, we conducted a series of paired experiments to test the effect of mutualism and the parasitoids on the oviposition preference of cotton leaf roller in a greenhouse (Fig. S1). Two plants were covered by a wooden cage with nylon net. Six 2^nd^ instar larvae of *S*. *derogata* were transferred to each cotton plant. Three comparisons were carried out to test whether mutualism would facilitate the host location of leaf roller, including one mutualism-present plant and a mutualism-absent plant (with only ants, only mealybugs or without ants and mealybugs). Each treatment was repeated for 10 times. The reconstructed ant colony was connected with the cotton plant by a silicone tube to allow the access of ghost ant to the plants. On mutualism-present plants, 60 mealybugs were inoculated into each plant beforehand. Two fertilized female *S*. *derogate*, *A. derogatae* and *A*. *bambawalei* were released on each plant after 24 h. We also tested the effects of mutualism on the oviposition of cotton leaf roller on plants with the exclusion of parasitoids. The number of leaf roller eggs and the parasitism by *A. derogatae* on each plant were counted and evaluated after one week.

### Effects of mutualism on interactions between *A. derogatae* and *S. derogate*

To test whether ant-mealybug mutualism influences the performance of *A. derogatae*, the method proposed by Errard and Hefetz^36^ was used to evaluate the aggressiveness between *A. derogatae* against *S*. *derogate* larvae. Two treatments were performed in this test, including the presence and absence of ant-mealybug mutualism. Ten shelters constructed by leaf roller larvae were transferred into a plastic case (25 × 18 × 12 cm). A cotton leaf with 30 3^rd^ instar nymph mealybugs was placed in the mutualism-present case, while a cotton leaf without mealybugs was transferred in the mutualism-absent case. The petiole of a cotton leaf was wrapped with moist cotton to maintain turgor. Twenty ghost ant workers and a female *A. derogatae* were also transferred into each case. The inner case was brushed with Teflon to prevent the ants and mealybugs from climbing out. The assigned *A. derogatae* female was allowed to acclimate for one minute in the plastic case before the test. Before hunting, *A*. *derogatae* was constantly observed to crawl on the cotton leaf. They would penetrate into cotton leaf slowly and tentatively with their ovipositors when they accessed the sites of leaf roller feeding. The parasitoids frequently waved their antennas and knocked on the leaf roller, and then bended their abdomen and fiercely penetrated into leaf roller larvae with their ovipositors when finding the leaf roller larvae. We scored the aggressive action of *A. derogatae* on leaf roller larvae in 5 minutes, including time for crawling around, tentative penetration, antenna knock, and fierce penetration, respectively. Aggressiveness of *A. derogatae* to leaf roller larvae was determined in both mutualism-present arena and mutualism-absent arena. Eight trials were conducted for each treatment. The aggressiveness index was calculated by using the following formula previously described by Errard and Hefetz^36^ (formula 1). The percentage of aggressiveness level was calculated and analyzed by formula 2.

1 $$\frac{{\sum }_{i=1}^{n}{\delta }_{i}{t}_{i}}{T}$$

2 $$\frac{{\delta }_{i}{t}_{i}}{{\sum }_{{\rm{i}}=1}^{n}{\delta }_{i}{t}_{i}}$$

*δ*_*i*_ represents the score we evaluated in the attack interaction. *t*_*i*_ means the duration of each behavioral interactions. *T* is the total time for each trial.

### Effects of patrolling ants on leaf roller oviposition

To determine the effect of ant tending on oviposition of leaf roller, the correlation between the number of patrolling ants and leaf roller oviposition was evaluated in field using cotton plants with similar size and growth. The selected plants were divided into two groups, one with the presence of ant-mealybug mutualism (n = 55), and the other with the absence of ant-mealybug mutualism (n = 70). Before the inoculation, the occasional caterpillars and eggs on plants were eliminated by banister brush. We transferred 60 3^rd^ instar mealybugs into each mutualism-present plant as described in the first experiment. Ghost ant patrolling was frequently observed on mealybug-infected plants. However, we did not perform inoculation of mealybugs in mutualism-absent cotton plants. The number of leaf roller eggs and foraging ants on plants were recorded after one week. The investigations were conducted on August 29^th^, 30^th^ and 31^st^, 2017. The average temperature was approximately 28 °C during the day.

### Effect of mutualism on spatial distribution of leaf roller eggs

Spatial distribution pattern was evaluated in both mutualism-present and mutualism-absent plants. Two cotton blocks were randomly selected, and each block was divided into 8 quadrats (15 × 15 m^2^). Thirty uniform cotton plants were randomly assigned into each quadrat. The quadrats were separated by 10–15 m from each other. The selected blocks were randomly used for one of two treatments, namely the presence and absence of the mutualism. Sixty 3^rd^ instar mealybugs were transferred into each cotton plant. To eliminate the effects of ant tending, the bases of the main stems of cotton plants were covered with paraffin to discourage ant approach and prevent mutualism prevalence in mutualism-absent plants. The numbers of leaf roller eggs and patrolling ants on plants were counted after one weeks. The investigation was conducted on September 7^th^, September 22^nd^, and October 9^th^, 2017, respectively.

### Statistical analysis

One-way analysis of variance (ANOVA) was used to compare the differences in means of leaf roller infection percentage and the number of eggs produced by leaf roller on plants. The number of eggs produced by leaf roller was log-transformed to satisfy the preconditions of variance analysis. Multiple comparisons of means were performed with Tukey HSD post-hoc analysis when the results of *F* test were significant. Paired sample t-test was performed to analyze the differences in the number of leaf roller eggs and the parasitism of leaf roller parasitoids in the paired test. Independent sample t-test was carried out to analyze the differences in aggressiveness index, aggressiveness level of *A*. *derogatae*, number of patrolling ants and leaf roller eggs between mutualism-present and mutualism-absent treatments. Linear regression model was used to determine the correlation between the number of patrolling ants and leaf roller oviposition in field. Iwao’s patchiness $(\mathop{x}\limits^{\ast }=a+\beta \overline{x})$ was used to analyze the spatial distribution pattern of leaf roller eggs and ant workers on plants. All statistical analyses were conducted with SPSS, version 19.0 (SPSS Inc., Chicago, IL, USA).

## Results

### Leaf roller infection and oviposition

**Figure 1:**
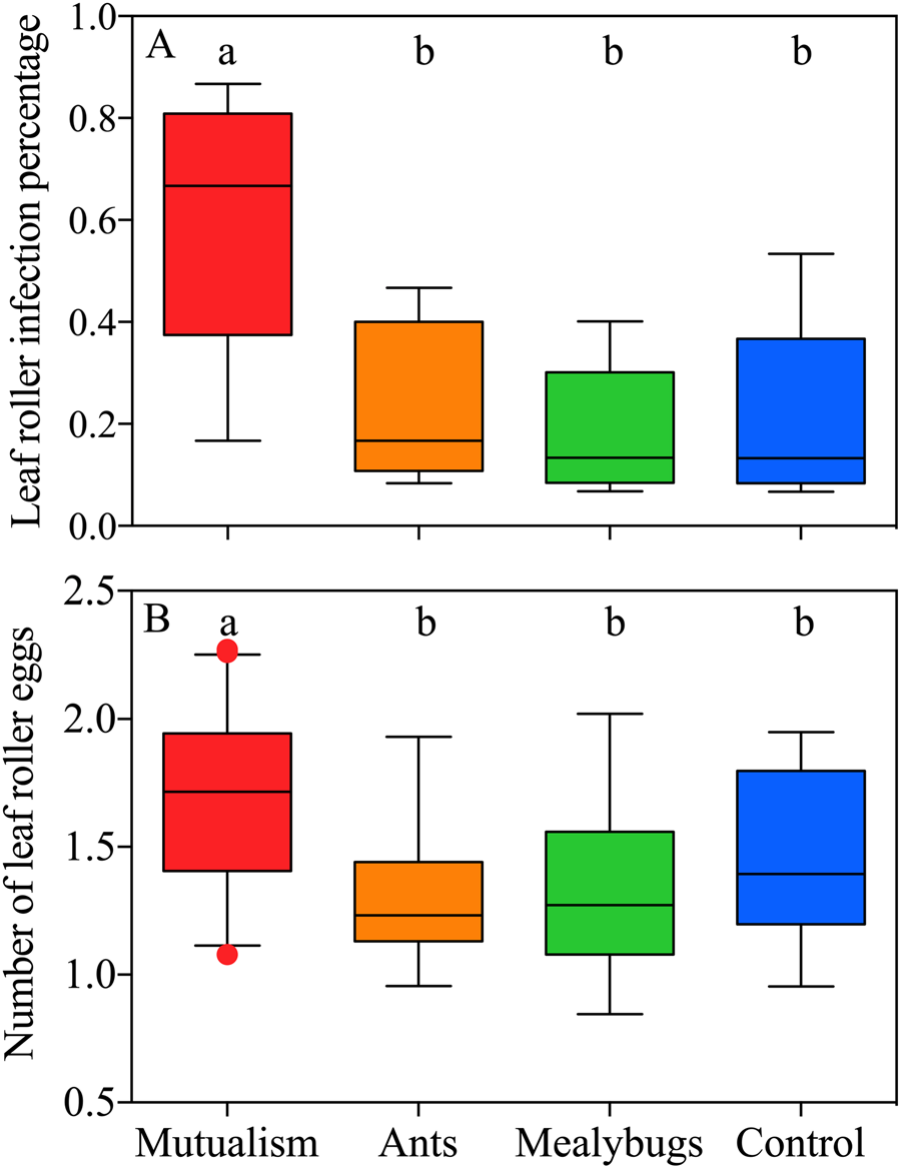
Effects of ant-mealybug mutualism on cotton leaf roller prevalence in field investigation. (**A**) Leaf roller infection percentage per plant; (**B**) leaf roller egg abundance per plant. Boxes extends from the 25^th^ to 75^th^ percentiles, with the band indicating median; whiskers represent the 5^th^ and 95^th^ percentiles; dots outside the whiskers are outliers. Boxes sharing the same letters indicate no significant differences among treatments (*P* > 0.05). The number of leaf roller eggs was log_10_ transformed.

Leaf roller infection on plants was significantly different among treatments (Fig. 1A; *F*_3, 16_ = 5.370, *P* = 0.009). Mutualism-present plants had a particularly higher oviposition preference of leaf roller than other plants (Fig. 1A; *P* = 0.037; *P* = 0.015; *P* = 0.023; respectively, Tukey HSD test). The number of leaf roller eggs was also significantly different among treatments (Fig. 1B; *F*_3, 79_ = 9.105, *P* < 0.001). Similarly, there were remarkably more leaf roller eggs on mutualism-present plants than on other plants (Fig. 1B; *P* < 0.001; *P* = 0.004; *P* = 0.007; respectively, Tukey HSD test).

**Figure 2:**
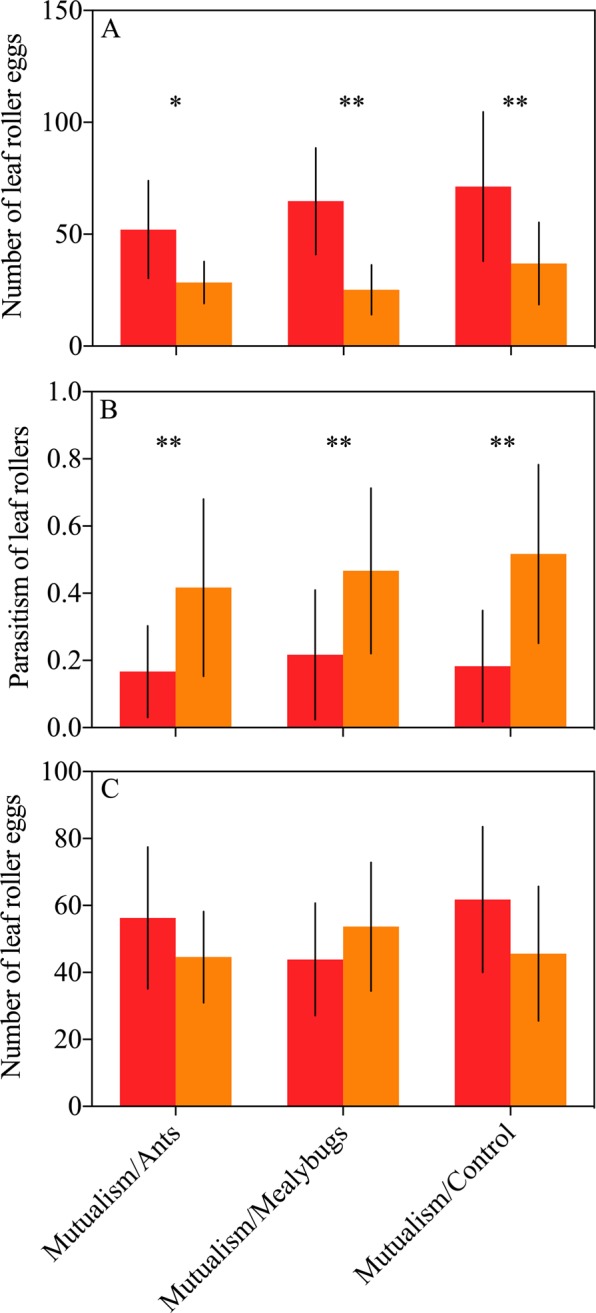
Effects of ant-mealybug mutualism on cotton leaf roller prevalence in greenhouse investigation. The data are presented as the mean ± SD. (**A**) leaf roller egg abundance per plant; (**B**) leaf roller larvae parasitism per plant; (**C**) leaf roller egg abundance without interference by *Apantetes derogatae*. Asterisk (*) and (**) on bars indicate significant differences between treatments (*P* < 0.05 and *P* < 0.01, respectively).

A significant difference was found in the number of leaf roller eggs in paired test. More leaf roller eggs were observed on mutualism-present plants than on other paired treatments (Fig. 2A; *t* = 3.005, *df* = 9, *P* = 0.015; *t* = 4.078, *df* = 9, *P* = 0.003; *t* = 3.324, *df* = 9, *P* = 0.01; respectively; paired sample *t* test). *Apantetes derogatae* parasitism was lower on mutualism-present plants than on other paired treatments (Fig. 2B; *t* = −3.503, *df* = 9, *P* = 0.007; *t* = −4.025, *df* = 9, *P* = 0.003; *t* = −3.586, *df* = 9, *P* = 0.006; respectively; paired sample *t* test). No pronounced difference in the number of leaf roller eggs was found between mutualism-present plants and other paired treatments when parasitoids were excluded (Fig. 2C; *t* = 1.294, *df* = 9, *P* = 0.228; *t* = −0.963, *df* = 9, *P* = 0.361; *t* = 1.403, *df* = 9, *P* = 0.194; respectively; paired sample *t* test).

### Parasitoid aggressiveness

**Figure 3:**
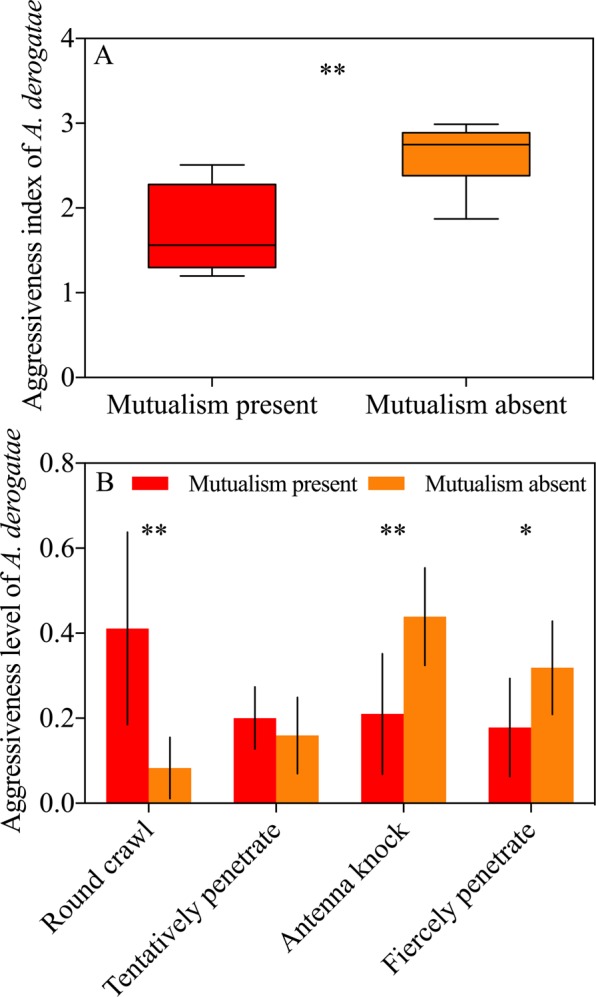
Effects of ant-mealybug mutualism on *Apantetes derogatae* aggressiveness. The data are presented as the mean ± SD. (**A**) Aggressiveness index; (**B**) percentage aggressiveness level. Boxes extends from the 25^th^ to 75^th^ percentiles, with the band indicating median; whiskers represent the 5th and 95th percentiles; Asterisk (*) and (**) on bars indicates significant differences between treatments (*P* < 0.05 and *P* < 0.01, respectively).

*Apantetes derogatae* showed significantly higher aggressiveness towards leaf roller in mutualism-absent arena than in mutualism-present arena (Fig. 3A, *t* = −4.118, *df* = 14, *P* = 0.001, independent sample t test). The percentage of time for crawling around of *A*. *derogatae* was much higher in mutualism-present arena (Fig. 3B, *t* = 3.910, *df* = 14, *P* = 0.002, independent sample t test). However, antenna knock and oviposition penetration of *A*. *derogatae* occurred more frequently in mutualism-absent arena (Fig. 3B, *t* = −3.594, *df* = 14, *P* = 0.003; *t* = −2.497, *df* = 14, *P* = 0.026, respectively; independent sample t test).

### Correlation between ant patrolling and leaf roller oviposition

**Figure 4:**
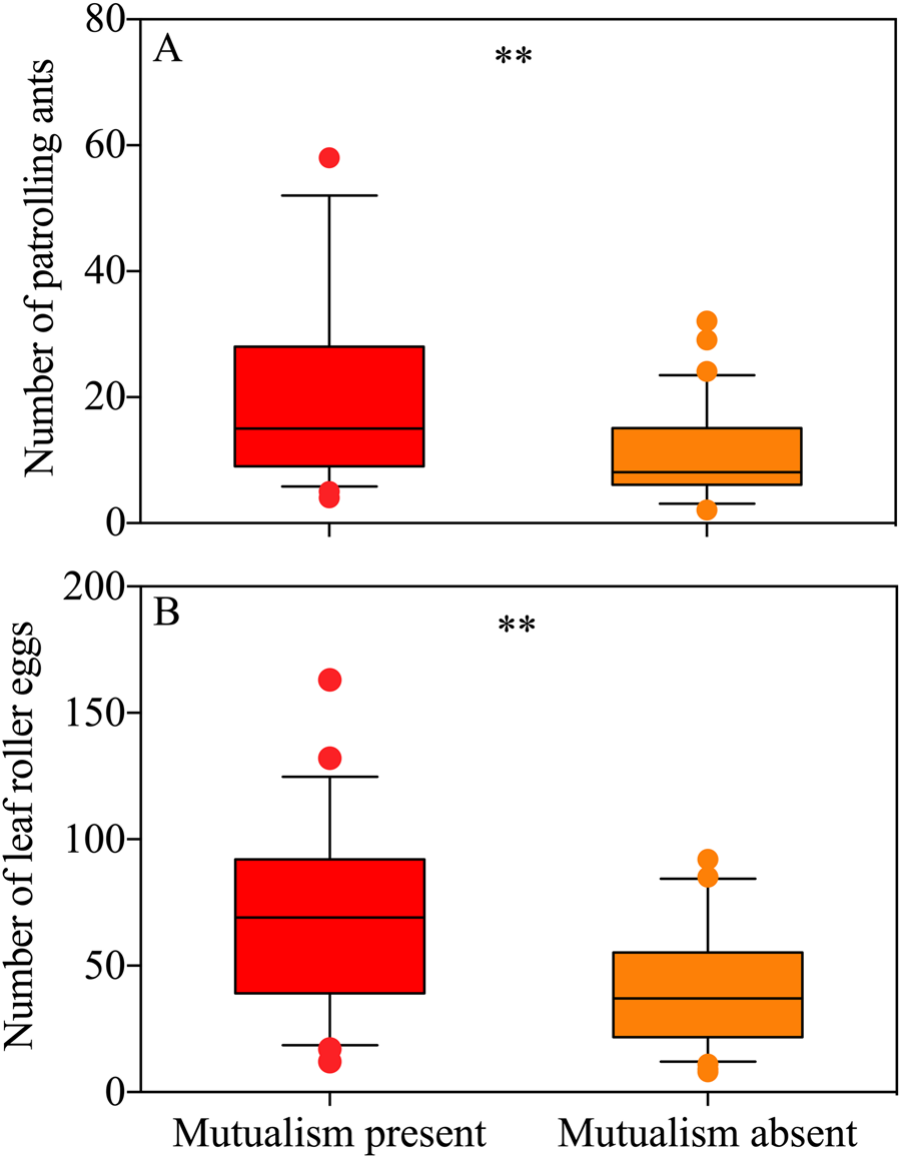
Effects of ant-mealybug mutualism on abundance of patrolling ants and leaf roller eggs on plants. (**A**) number of patrolling ants; (**B**) number of leaf roller eggs. Boxes extends from the 25^th^ to 75^th^ percentiles, with the band indicating median; whiskers represent the 5th and 95th percentiles; dots outside the whiskers are outliers. Asterisk (**) on boxes indicates significant differences between ant-mealybug mutualism presence and absence (*P* < 0.01).

**Figure 5:**
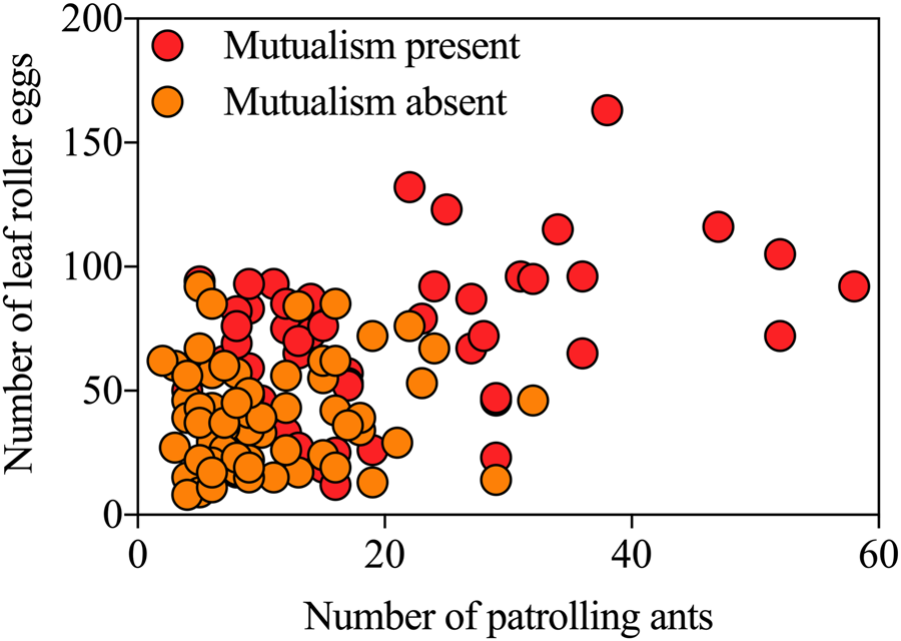
Effects of ant-mealybug mutualism on correlation between abundance of leaf roller eggs and patrolling ants. Red circle: mutualism presence; orange circle: mutualism absence.

The number of patrolling ants on mutualism-present plants was significantly larger than that on mutualism-absent plants (Fig. 4A; *t* = 4.945, *df* = 123, *P* < 0.001, independent sample t test), and it was the same case for the number of leaf roller eggs (Fig. 4B; *t* = 5.984, *df* = 123, *P* < 0.001, independent sample t test). There was an obvious liner correlation between the number of patrolling ants and leaf roller oviposition on mutualism-present plants (Fig. 5; *y* = 45.595 + 1.127*x*; *R*^2^ = 0.217; *F*_1, 53_ = 14.667, *P* < 0.001; red circle). However, no significant liner correlation was found on mutualism-absent plants (Fig. 5; *y* = 34.780 + 0.407*x*, *R*^2^ = 0.016; *F*_1, 68_ = 1.110, *P* = 0.296; orange circle).

### Spatial distribution pattern of leaf roller eggs

**Figure 6:**
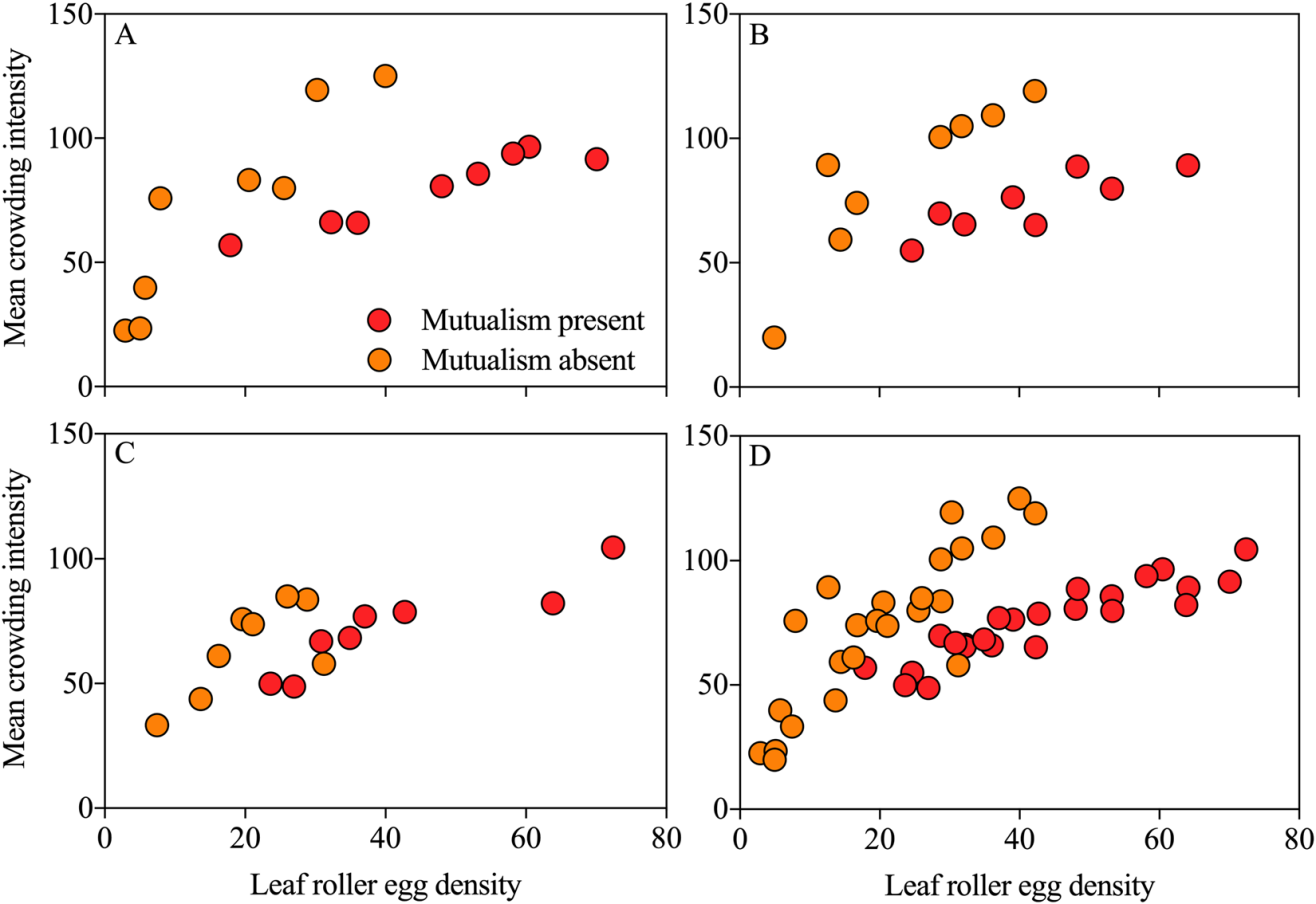
Effects of ant-mealybug mutualism on spatial distribution pattern of leaf roller egg. (**A**) Sep.7; (**B**) Sep.22; (**C**) Oct.9; (**D**) superimposed data set from Sep.7 to Oct.9. Red circle: mutualism presence; orange circle: mutualism absence.

The results revealed a spatially aggregated distribution pattern of leaf roller eggs at the absence of ant-mealybug mutualism (Orange circle: Fig. 6A, Sep.7: $\mathop{x}\limits^{\ast }=25.241+2.663\overline{x}$, *R*^2^ = 0.853, *P* = 0.001; Fig. 6B, Sep.22: $\mathop{x}\limits^{\ast }=32.470+2.220\overline{x}$, *R*^2^ = 0.796, *P* = 0.003; Fig. 6C, Oct.9: $\mathop{x}\limits^{\ast }=29.742+1.685\overline{x}$, *R*^2^ = 0.523, *P* = 0.043). However, the distribution of leaf roller eggs was uniform at the presence of ant-mealybug mutualism (Red circle: Fig. 6A, Sep.7: $\mathop{x}\limits^{\ast }=40.716+0.829\overline{x}$, *R*^2^ = 0.914, *P* < 0.001; Fig. 6B, Sep.22: $\mathop{x}\limits^{\ast }=41.722+0.768\overline{x}$, *R*^2^ = 0.721, *P* = 0.008; Fig. 6C, Oct.9: $\mathop{x}\limits^{\ast }=33.082+0.938\overline{x}$, *R*^2^ = 0.829, *P* = 0.002). We also superposed the data from Sep.7 to Oct.9 and analyzed the spatial distribution patterns in a superimposed data set. Leaf roller eggs also showed a significantly aggregated or a uniform distribution pattern at the presence or absence of ant-mealybug mutualism (Fig. 6D, orange circle: $\mathop{x}\limits^{\ast }=25.903+2.325\overline{x}$, *R*^2^ = 0.748, *P* < 0.001; red circle: $\mathop{x}\limits^{\ast }=37.418+0.869\overline{x}$, *R*^2^ = 0.830, *P* < 0.001).

### Spatial distribution pattern of ants

**Figure 7:**
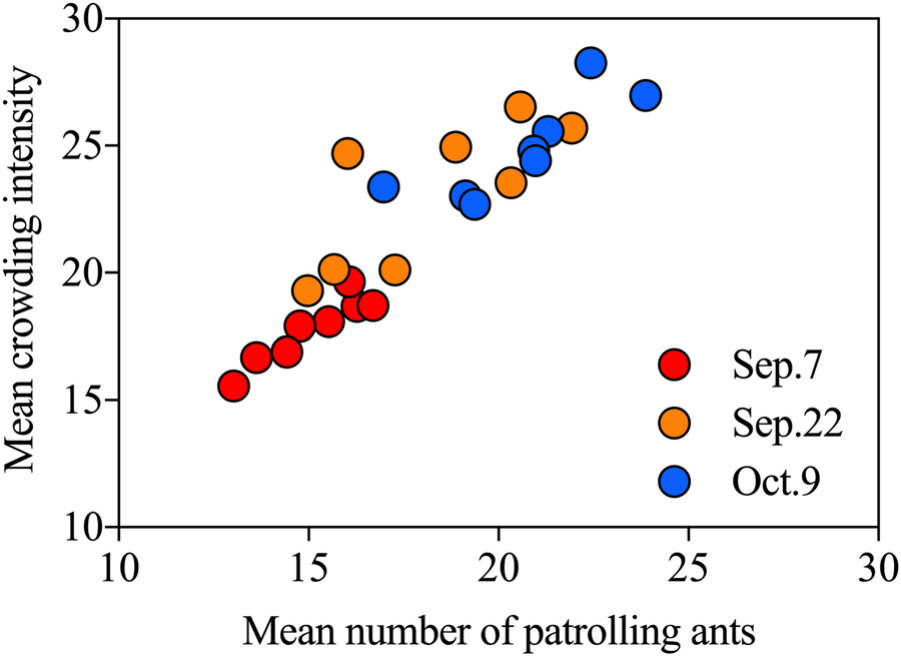
Spatial distribution pattern of ant workers on plants. Red circle: Sep.7; orange circle: Sep.22; blue circle: Oct.9.

**Figure 8:**
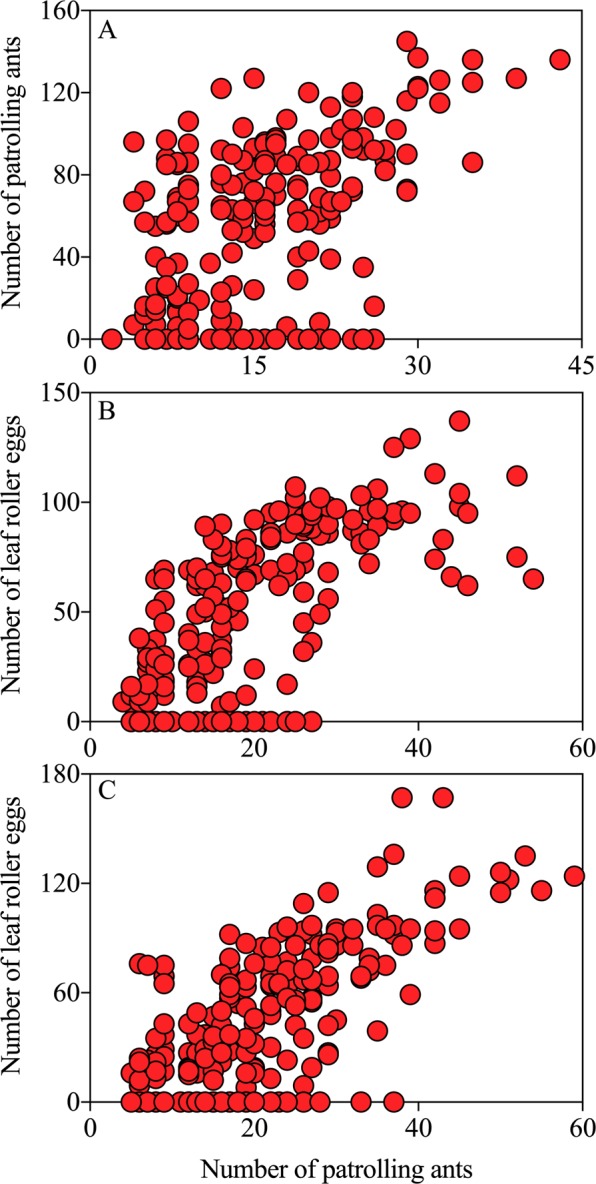
Correlation between abundance of leaf roller eggs and patrolling ants on plants. (**A**) Sep.7; (**B**) Sep.22; (**C**) Oct.9.

Ant workers showed a consistently uniform distribution pattern on plants on three investigation dates (Fig. 7; Sep.7, $\mathop{x}\limits^{\ast }=3.675+0.936\overline{x}$, *R*^2^ = 0.861, *P* = 0.001; Sep.22, $\mathop{x}\limits^{\ast }=7.956+0.833\overline{x}$, *R*^2^ = 0.573, *P* = 0.03; Oct.9, $\mathop{x}\limits^{\ast }=9.122+0.765\overline{x}$, *R*^2^ = 0.686, *P* = 0.011). Significant correlations were also observed between the abundance of patrolling ants and the number of leaf roller eggs on plants on three investigation dates (Fig. 8A; Sep.7, *y* = 4.226 + 2.842*x*, *R*^2^ = 0.258, *F*_1, 238_ = 82.596, *P* < 0.001; Fig. 8B; Sep.22, *y* = −4.261 + 2.518*x*, *R*^2^ = 0.490, *F*_1, 238_ = 228.213, *P* < 0.001; Fig. 8C; Oct.9, *y* = −12.605 + 2.626*x*, *R*^2^ = 0.493, *F*_1, 238_ = 231.008, *P* < 0.001).

## Discussion

Clarification of the link between environmental change and ecosystem structure and function is pivotal to revealing the complex mechanisms of multitrophic interactions, which largely influence the connection in the food webs of ecosystems^37^. Compared with competition and predation, little is known about the effects of mutualistic interactions on the function and stability of local ecosystems. Ant-hemipteran mutualism commonly occurs in wide geographic regions from temperate to tropical latitudes^3,38^. However, there is a poor understanding of the effects of ant-hemipteran mutualism on complex trophic interactions and local community structure. In order to understand the complex evolution of ecosystems, it is highly necessary to explore the biological and ecological mechanisms that play key roles in generating and mediating the context-dependent food webs in ecosystems.

Due to their biological characteristics and social behaviors, omnivorous ants possess efficient recruitment and strong aggressiveness, and can develop abundant population and territoriality. Omnivorous ants can encounter and interact with almost any other insect group and have strong direct or indirect effects on terrestrial food webs, which may positively or negatively influence local community^8,39^. Some invasive ant species become more aggressive towards other arthropods when they assimilate the honeydew produced by hemipterans^9,40^, aggravating the effects of dominant ants on local food webs. The stimulation of ants by honeydew-producing hemipterans may elevate and diversify the effects of ants on plant-based arthropod food webs^40,41^. The cascading effects of ant-hemipteran mutualism on food webs are complex and highly context-dependent. Ant-aphid mutualism facilitates plant reproduction and changes the trophic structure because it can reduce caterpillar survival and the damage of caterpillar herbivores on plants^24^. However, ants can also cause potential risks to plants, because they are often associated with local hemipteran outbreaks, and an increased density of honeydew-producing hemipterans will exacerbate plant sap consumption and pathogen transmission by hemipterans^42,43^.

*Tapinoma melanocephalum* has a wide distribution and is highly competitive in sugar resource against sympatric ant species. Increasing studies have explored the reciprocal effects between *T. melanocephalum* and mealybugs^29–31,44^. However, the outcomes at the community level are largely ignored. Our results present the novel effects of ant-mealybug mutualism on the co-occurring herbivores and parasitoids. We found that cotton plants with ant-mealybug mutualism are more attractive sites for leaf roller oviposition. Patrolling ants frequently protect leaf roller from the attack by *A. derogatae*. Ants can effectively utilize the shelters constructed by leaf roller larvae as a refuge for mealybugs^35^. However, withdrawal of ant-mealybug mutualism would largely reduce the benefits of ant tending for leaf roller adults and larvae. These results suggest a reciprocal mutualism can be formed among ant, mealybug and cotton leaf roller (Fig. S2). Due to the consumption of herbivores by ants, an antagonistic interaction between ant and co-occurring herbivores is commonly observed in ant-plant mutualism^45–47^. However, we found an extraordinary synergism between ant-mealybug mutualism and leaf roller on cotton plants. Besides, there was a significant correlation in the abundance of leaf roller eggs and patrolling ants on plants, which also demonstrates the interdependent association between ants and leaf roller.

Compared with on mutualism-present plants, *A. derogatae* showed higher parasitism and aggressiveness towards leaf roller on mutualism-absent plants. These results indicate that mutualism prevalence on the plants could improve the fitness of leaf roller and suppress the performance of *A. derogatae*. Cotton plants hosting mealybugs have an indirect positive effect on leaf roller by enhancing ant patrolling on the plants. Exclusion of mealybugs from the plants interrupted the reciprocal association and changed the foraging behavior of *T. melanocephalum*. There was a definite decline of ant patrolling activity on mealybug-excluded plants. The significant correlation between the abundance of leaf roller eggs and patrolling ants disappeared when mealybugs were absent. These results suggest that the limited amounts of carbohydrates provided by mealybugs play a key role in maintaining the mutualism stability. Previous studies have also clarified the role of honeydew produced by hemipterans both as a source of energy and as a mediator in ant-hemipteran interactions^48,49^. Our results showed the cascading effects triggered by manipulation of trophobionts on the context-dependent trophic structure.

Antagonistic interactions, such as predation and parasitism, may have direct or indirect effects that may trigger extensive changes in community composition and stability^6^. Organism communities can be affected by predator effects^50^. Herbivores are suppressed by predators, which can be largely attributed to the consumption of herbivores and reshaping of herbivore behavior and distribution^45^. Predaceous ant, *Forelius pruinosus*, consumes and disturbs a dominant lepidopteran folivore, *Bucculatrix thurberiella* (Bucculatricidae); in cotton plants with ant-plant mutualism, ants could alter the spatial distribution of both caterpillars and their damage^45^. Our results show the positive effects of the ant-mealybug interaction on the local herbivore community. Ant-mealybug mutualism not only increased the leaf roller prevalence in cotton field, but also reduced the parasitism performance of the parasitoids and shaped the spatial distribution of ambient herbivores. We speculate that *S. derogate* modulates its own spatial distribution pattern in mutualism-present arena in response to the protective services provided by patrolling ants, and the distribution pattern of reciprocal ants may shape the distribution characteristics of plant herbivores. We observed a consistently uniform distribution pattern of ant workers on plants. Prevalence of leaf roller may cause a pronounced decrease in plant growth and reproduction because of its atrocious polyphagy. However, ant-aphid mutualism is considered to be beneficial for plant fitness because ants disturb the consumption of plants by caterpillars, which causes relatively more serious damage to the host plant compared aphids^24^. Ant-aphid mutualism is associated with herbivory decrease, which was also presented by other studies^13,15^. Our results highlight the potential benefits of ant-mealybug mutualism to surrounding herbivores owing to promoted anti-parasitoid effects.

By examining the effect of mutualism between ants and invasive mealybugs, our results reveal the significance of mutualistic interactions in shaping the pattern of certain key ecological processes, including ant abundance, herbivore population and distribution, and parasitoid performance. Considering that *S. derogate* and *A. derogatae* are respectively important herbivores and parasitoids in cotton in our study, the ant-mealybug mutualism may have more trophic cascading effects on local community composition and structure. More studies of other mutualistic systems should be conducted to facilitate a better understanding of the overall effects of mutualistic interactions on the surrounding arthropod community.

### Ethical approval

This article does not contain any studies with human participants or vertebrate performed by any of the authors.

## Supplementary information

Supplementary Information
