# Supplementary material for: Ant-mealybug mutualism modulates the performance of co-occurring herbivores
Source: Sci Rep. 2019 Sep 10;9:13004. doi: 10.1038/s41598-019-49334-3 (PMC6737150; doi:10.1038/s41598-019-49334-3)
Supplement: Supplementary file 1 — Supplementary Information [file 41598_2019_49334_MOESM1_ESM.pdf]

Ant-mealybug mutualism modulates the performance of co-occurring herbivores

Chong Xu[1#], Jia Su[1#], Xiaobin Qu[1], Aiming Zhou[1*]
1 Hubei Insect Resources Utilization and Sustainable Pest Management Key Laboratory, College of Plant Science and Technology, Huazhong Agricultural University, Wuhan, 430070, China
*Corresponding author's e-mail address:
zhouam@mail.hzau.edu.cn;
Author information
Corresponding author: Aiming Zhou;
Department of Hubei Insect Resources Utilization and Sustainable Pest Management Key Laboratory, College of Plant Science and Technology, Huazhong Agricultural University, Wuhan, 430070, People's Republic of China
E-mail address: zhouam@mail.hzau.edu.cn

FIGURE LEGENDS

Figure S1 Graphical representation of apparatus used in laboratory test. Two cotton plants were covered by a wooden cage. One plant received ant tending but another did not. Leaf roller larvae damaged cotton leaves and constructed shelters for mealybugs.

Figure S2 Graphical representation of mutualism and antagonism in trophic cascade. *Sylepta derogate* preferred to lay eggs on ant-mealybug mutualism present plants. Leaf roller larvae provided shelters for mealybugs from parasitism by *A. bambawalei*, and ants protected leaf roller larvae from parasitism by *A. derogatae*. Reciprocal interactions were established among ant, mealybug, and cotton leaf roller in cotton field.

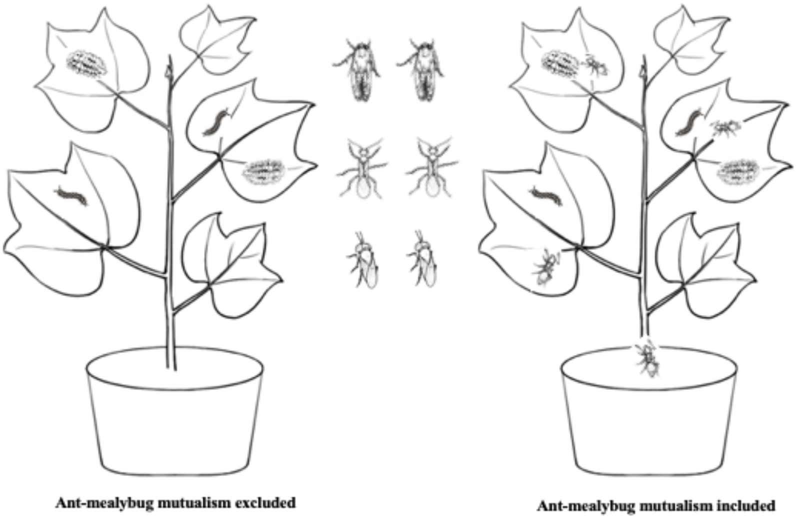
Ant-mealybug mutualism excluded
Ant-mealybug mutualism included

Figure S1

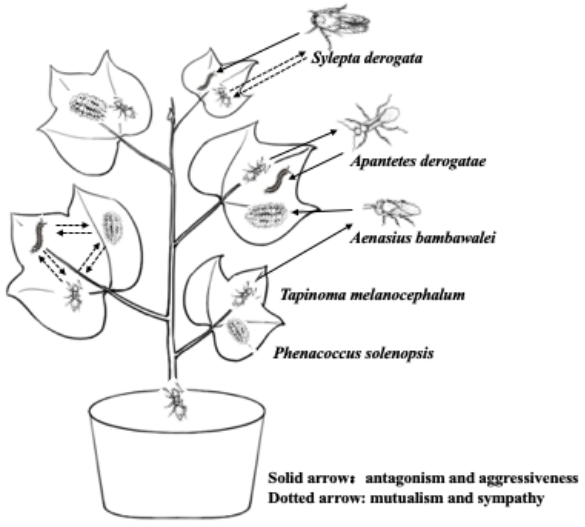
Sylepta derogata
Apantetes derogatae
Aenasius bambawalei
Tapinoma melanocephalum
Phenacoccus solenopsis
Solid arrow： antagonism and aggressiveness
Dotted arrow: mutualism and sympathy

Figure S2
